# Trauma recidivism at an emergency department of a Swedish medical center

**DOI:** 10.1186/s40621-016-0087-2

**Published:** 2016-09-12

**Authors:** Fredrik Röding, Marie Lindkvist, Ulrica Bergström, Olle Svensson, Jack Lysholm

**Affiliations:** 1 Division of Surgery and Perioperative Sciences, Department of Orthopaedics, Umea University, 90187 Umea, Sweden; 2 Department of Public Health and Clinical Medicine, Epidemiology and Global Health, Umea University, 90187 Umea, Sweden; 3 Umea School of Business and Economics, Department of Statistics, Umea University, 90187 Umea, Sweden; 4 Department of Public Health and Clinical Medicine, Epidemiology and Global Health, Centre of Quality Registries North Sweden, Umea University, 90187 Umea, Sweden

**Keywords:** Descriptive epidemiology, Trauma, Recidivism, Longitudinal

## Abstract

**Background:**

To inform targeted prevention, we studied patterns of trauma recidivism and whether a first injury predicts the risk for a recurrent injury.

**Methods:**

In a population-based study of 98,502 adult injury events 1999–2012, at the emergency department of Umeå University Hospital, Sweden, we compared non-recidivists with recidivists in terms of patients’ sex, age, type of injury and severity of the injury.

**Results:**

Thirty-six percent of all patients suffered recurrent injuries, which were associated with a higher proportion of inpatient care and more hospital days. Young men and elderly women were at the highest risk for trauma recidivism. At 20 to 24 years, men had a 2.4 (CI 95 % 2.3–2.5) higher risk than women, a 90 years old woman had almost a 10-fold higher risk for another moderate/severe injury than a 20 years old one. A fracture were associated with a hazard ratio of 1.28 (CI 95 % 1.15–1.42) among men younger than 65 years and 1.31 (CI 95 % 1.12–1.54) for men older than 65 years for a subsequent moderate/severe injury. For women younger than 65 years a fracture was associated with a hazard ratio of 1.44 (CI 95 % 1.28–1.62) for a subsequent moderate/severe injury. A sprain carries a higher risk for a new moderate/severe injury for both men and women and in both age groups; the hazard ratio was 1.13 (CI 95 % 1.00–1.26) for men younger than 65 years, 1.42 (CI 95 % 1.01–1.99) for men older than 65 years, 1.19 (CI 95 % 1.05–1.35) for women younger than 65 years and 1.26 (CI 95 % 1.02–1.56) for women older than 65 years. A higher degree of injury severity was associated with a higher risk for a new moderate/severe injury.

**Conclusion:**

Trauma recidivism is common and represents a large proportion of all injured. Age and sex are associated with the risk for new injury. Injury types and severity, also have implications for future injury.

## Background

Injury is a major public health problem. In the European Union (EU), 230,000 trauma casualties are recorded annually and everyday more than 112,000 people are admitted to hospital for injuries (Bauer et al. 2014). In the Unites States (US), injury is the dominant killer below the age of 46 (Rhee et al. 2014). But not only severe injuries put a burden of injury on society. Minor and moderate ones are more common and, even if less severe, this injuries account for more than a third of the burden of injuries according to Disability-adjusted life years (DALYs) (Polinder et al. 2012). Improvements in treatment and prevention of cancer and cardiovascular disease during recent decades have left trauma as a proportionally more important cause to the total burden of ill health.

Traditionally injuries have been regarded as occurring by chance and thus not possible to prevent. But injury incidence is not randomly distributed e.g., boys are more injury-prone than girls and elderly women are more affected by injury than elderly men (Saveman and Bjornstig 2011; Hedstrom et al. 2012). We also know that social deprivation, substance abuse and mental illness increase the risk for injury (Gentilello et al. 1995; Wan et al. 2006; Fleury et al. 2010).

Emergency department staff know their recidivist patients. Reiner et al. surveyed ED staff and found that trauma recidivists more often were male, young and under the influence of alcohol or drugs, and introduced the concept of trauma recidivism (Reiner et al. 1990). Several studies have confirmed these results, (Poole et al. 1993; Hedges et al. 1995; Williams et al. 1997; Sayfan and Berlin 1997; Kaufmann et al. 1998; Keough et al. 2001; Caufeild et al. 2004; Worrell et al. 2006; Brooke et al. 2006; Claassen et al. 2007; Toschlog et al. 2007; Kwan et al. 2011; Allan et al. 2013; Rittenhouse et al. 2015). As a response, several trauma centers developed intervention programs. Brief interventions shortly after the injury have been shown to halve the risk of renewed alcohol-related injury (Schermer et al. 2006; Gmel et al. 2007). Violence intervention programs has showed a fourfold to sixfold decrease of trauma recidivism and with good cost-effectiveness (Cooper et al. 2006; Smith et al. 2013; Juillard et al. 2015). Other examples of successful intervention are fall prevention in geriatric care and fracture prevention programs (Lih et al. 2011; Sach et al. 2012; Van der Kallen et al. 2014). Intervention programs seem to working better if implemented shortly after the injury event (Gentilello et al. 1995; Smith et al. 2013).

For targeted prevention knowledge about risk factors is crucial. Most studies on trauma recidivism regard cases of severe injury from urban level 1 trauma US hospitals. There is a lack of knowledge on other aspects on trauma recidivism as only a few studies address trauma recidivism in a rural populations and a wider age perspective. These studies indicate that female sex and older age may be risk factors for trauma recidivism (Williams et al. 1997; Toschlog et al. 2007). Another rural population study showed a recidivism rate of 12 % during just 1-year follow up. The fact that not only severe injuries contribute to the burden of ill health, emphasizes the need for studies, which also including less severe injuries, since these are more common.

Our objective was to estimate the burden of trauma recidivism in a stable population including less severe injuries and to investigate whether certain conditions associated with an injury are risk factors for a subsequent injury.

## Methods

### Study design

A retrospective, population-based, longitudinal study on acute adult trauma, 1999–2012.

### Material

Since 1993, almost all trauma emergencies handled at Umeå University Hospital are registered in an injury database. The register is part of the European Injury Database (IDB). (http://www.socialstyrelsen.se/register/halsodataregister/patientregistret/idbsverige). The hospital is responsible for all emergency medical services for the Umeå region (*n* = 145,000), Northern Sweden. The population consists of the residents in the A-64 region registered by the Swedish Tax Agency associated with the municipalities Umeå, Robertfors, Vindeln, Vännäs, Bjurholm and Nordmaling. Only residents registered by the Swedish Tax Agency were included in the study. (The inhabitants of Sweden are obliged by law to inform the Tax Agency about their address of residence within 1 week after moving to a new area) and all visitors from others parts of Sweden or other countries were excluded. In the emergency room, the patient or a proxy fills in a voluntary questionnaire about the circumstances around the injury. If needed, the injury surveillance group at the hospital supplements the information from medical files, ambulance records and police reports. Annually, about 7300 cases per year were recorded. The database was validated every year for missing cases by the hospital’s patient registry for inpatient cases and by billing information from the Emergency Department for outpatient cases. There were 2.3 % missing cases.

### Variables

We merged injury types into ten main groups; fracture, contusion, wound, sprain, concussion, foreign body, thermo, strain, poisoning and other. The Injury Severity Score (ISS) was used: 1–3 for mild, 4–8 for moderate, and ≥9 for severe injuries (Baker et al. 1974). The ISS is based on the AIS (abbreviated injury scale) (Association for the Advancement of Automotive Medicine 2001), a 6-grade scale, where AIS 1 is a mild injury and AIS 6 denotes a fatal one. An example of AIS 1 is a superficial wound and AIS 3 a femur fracture. The three most severely injured body regions have their AIS number squared and added up to give the ISS score. Recurrent moderate injury was defined as a recurrent injury at least ISS 4 level. This study was approved by the Regional Ethical Review Board in Umeå (2013-61-31 M). A recurrent injury was defined as a new injury after a first injury during the study period.

### Statistical methods

Incidence of the first and recurrent injury was calculated by dividing the number of injuries by the average midyear population for the A-64 region for each age group during this period (Statistics Sweden). Incidence rate ratios were calculated with 95 % confidence interval. Analyses of mean values (age and mean hospital days) were made with two-sided independent t-test and for comparison of proportions (sex and proportion outpatient/in-hospital) chi-squared test was used. To investigate the association between independent variables (age, injury types and injury severity) and risk of recurrent moderate or severe injury, Cox regression analysis was used. For injury types each category was controlled against all other categories as the reference. An individual with no recurrent injury within 5 years was considered censored. The data of the database were supplemented with death date from the tax agency register. Multiple Cox regression analysis was used to analyze association and to adjust for age as a confounding factor, at a rejection level of 5 %.

## Results

Of the 98,502 injuries recorded, 37,396 persons were injured once and 21,526 (36 %) twice or more. Men more frequently suffered singular acute trauma as well as repetitive acute trauma. Men was also relatively more common among trauma recidivists. The mean age at the first injury was lower for men and higher for women in the trauma recidivist group compared to non-recidivists (Table 1). Recurrent injuries required more in-hospital care and more hospital days (Table 2). ISS 1–3 represented 70 % of all injuries, ISS 4–8 24 % and ISS ≥9 6 %.

**Table 1:** Descriptive data for non-recidivists and recidivists, year 1999 to 2012

	Non-recidivists	Recidivists	*P*-value
*Individuals*	37,396 (63.5 %)	21,526 (36.5 %)	
*Sex*			
Men	19,358 (51.8 %)	12,069 (56.1 %)	0.001
Women	18,038 (48.2 %)	9,457 (43.4 %)	0.001
*Age (y), mean ± SD*			
Men	43.5 year ± 19.3	41.3 year ± 18.7 ^a^	0.001
Women	47.9 year ± 21.3	53.2 year ± 22.0 ^a^	0.001

^a^ Age at the first injury

**Table 2:** Descriptive data for first injury and the recurrent injury, year 1999 to 2012

	First injuries	Recurrent injuries	*P*-value
*Number*	58,922 (59.8 %)	39,580 (40.2 %)	
Proportion outpatient/in-hospital	82.9 %/17.1 %	78.5 %/21.5 %	0.001
Hospital days *mean ± SD*	9.7 ± 17.0	10.6 ± 16.4	0.001
Total hospital days	97,768 (52 %)	90,271 (48 %)	
*Injury types*			
Fracture	14,439 (24.5 %)	9,728 (24.6 %)	
Contusion	12,351 (21.0 %)	9,402 (23.8 %)	
Wound	13,039 (22.1 %)	8,692 (22.0 %)	
Sprain	10,648 (18.1 %)	5,998 (15.2 %)	
Concussion	1,862 (3.2 %)	1,161 (2.9 %)	
Foreign body	1,420 (2.4 %)	1,283 (3.2 %)	
Thermo	730 (1.2 %)	465 (1.2 %)	
Overstrain	650 (1.1 %)	448 (1.1 %)	
Poison	541 (0.9 %)	805 (2.0 %)	
Other	3,242 (5.5 %)	1,598 (4.0 %)	

### Age and sex

**Fig. 1:**
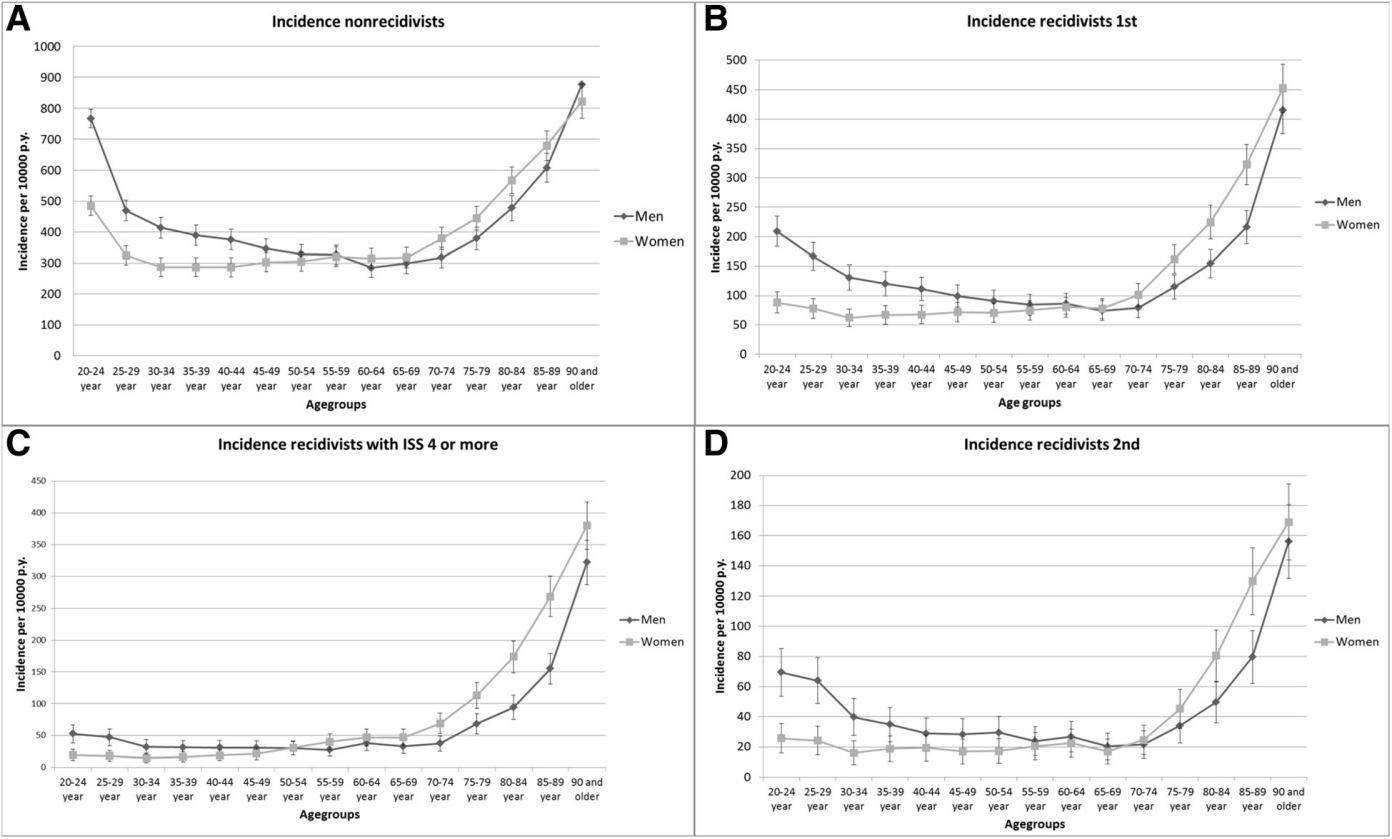
Injury incidence for women and men in 5 years age groups with 95 % confidence interval for **a** Non recidivist **b** 1st recidivists **c** 1st recidivists with ISS 4 or more and **d** 2nd recidivists. Year period 1999–2012

**Fig. 2:**
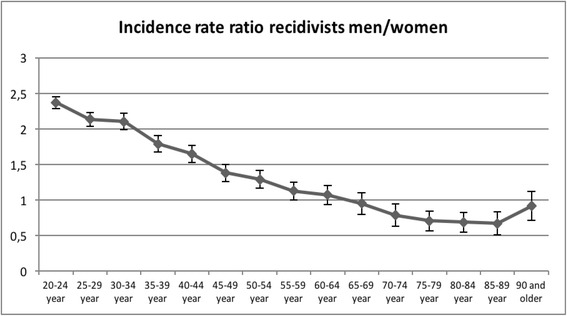
Incidence rate ratio for men/women with 95 % confidence interval for 1st recidivists. Year period 1999–2012

Young and old people were most injury-prone, men more than women until the age of 50, when incidence equalized. For recurrent injuries were the men/women ratio was even more pronounced (Fig. 1). For the youngest age group (20–24 years), men were 2.4 (CI 95 % 2.3–2.5) times more likely than women to sustain a first injury and 2.7 (CI 95 % 2.6–2.8) times more likely to sustain a second recurrent injury (Fig. 2). From the age of 70 women suffered more recurrences.

The risk for a recurrent injury with moderate or severe outcome (ISS 4 or more), within 5 years from the first injury, showed that the risk was lowest among women 20 to 29 years old and increased with age. A 90-year-old woman had a 9.7 times higher hazard rate for recurrent moderate injury than a 20-year-old woman. The lowest hazard rate for men was found in the age group 30–49 years and increased with higher and as well as lower age (Table 3).

**Table 3:** Hazard ratio for moderate ^a^ second injury in relation to different age groups (age at first injury). The analyses were divided by gender

	Men			Women		
	HR	95 % CI		HR	95 % CI	
Age groups, years						
20–29 year	1.00			1.00		
30–39 year	0.76	0.68	0.86	ns		
40–49 year	0.82	0.72	0.93	1.39	1.18	1.64
50–59 year	ns ^b^			2.09	1.81	2.42
60–69 year	ns			2.73	2.36	3.16
70–79 year	1.89	1.65	2.16	4.82	4.22	5.50
80–89 year	3.00	2.58	3.48	7.20	6.32	8.22
90 and older	6.04	4.50	8.11	9.66	7.97	11.72

^a^ Moderate is defined as an injury with at least ISS 4 level

^b^ *ns* not significant

### Injury type

Fractures and sprains in men increased the hazard rate for future moderate or severe injury (ISS ≥4) more than for other injury types while wounds as well as foreign body injuries (mainly corneal lesions) did not. Women had a similar pattern except that fractures did not increase the hazard rate in the older age group. Younger men with concussion as first injury had a higher hazard for future moderate or severe injury (Table 4).

**Table 4:** Hazard ratio for moderate ^a^ second injury in relation to first injury type. The analyses were divided by gender and age group and adjusted for age (in years) within each age group. The reference is all other injuries

	Men				Women			
Age group	20–65 year		66 year and older		20–65 year		66 year and older	
	HR	95 % CI	HR	95 % CI	HR	95 % CI	HR	95 % CI
Concussion	1.41	(1.12–1.77)	ns ^b^		ns		ns	
Contusion	ns		ns		ns		ns	
Fracture	1.28	(1.15–1.42)	1.31	(1.12–1.54)	1.44	(1.28–1.62)	ns	
Sprain	1.13	(1.00–1.26)	1.42	(1.01–1.99)	1.19	(1.05–1.35)	1.26	(1.02–1.56)
Wound	0.89	(0.80–0.98)	0.78	(0.63–0.95)	0.84	(0.72–0.97)	0.83	(0.70–0.98)
Poison	ns		ns		ns		ns	
Thermo	ns		ns		ns		ns	
Foreign body	0.76	(0.59–0.98)	ns		0.44	(0.23–0.84)	ns	

^a^ Moderate is defined as an injury with at least ISS 4 level

^b^ *ns* not significant

### Injury severity

Severe and moderate injuries among women resulted an increased hazard rate for a subsequent moderate or severe injury compared to mild injuries. Men had a similar pattern although severe injuries did not increase the hazard rate for a subsequent moderate or severe injury among old men and moderate injuries did not increase the hazard rate among men younger than 65 years old (Table 5).

**Table 5:** Hazard ratio for moderate ^a^ second injury in relation to first injury level of severity. The analyses were divided by gender and age group and adjusted for age (in years) within each age group

	Men				Women			
Age group	20–65 year		66 year and older		20–65 year		66 year and older	
	HR	95 % CI	HR	95 % CI	HR	95 % CI	HR	95 % CI
ISS 1–3	1.00		1.00		1.00		1.00	
ISS 4–8	1.46	(1.31–1.63)	ns ^b^		1.49	(1.32–1.69)	1.16	(1.03–131)
ISS 9 or more	ns		1.41	(1.10–1.81)	2.14	(1.53–2.98)	1.25	(1.06–1.46)

^a^ Moderate is defined as an injury with at least ISS 4 level

^b^ *ns* not significant

## Discussion

### Proportion

Trauma recidivists account for 36 % of all injured, a higher proportion than found in most other studies. This can be explained by a 14 year follow up which is longer than in most other studies. Moreover, data were collected from the only responsible trauma hospital in the region and from a stable population. Previous studies present a great variety of the contribution of trauma recidivism to the injury burden. The majority of these studies are based on data from major trauma centers in the US (Poole et al. 1993; Hedges et al. 1995; Kaufmann et al. 1998; Brooke et al. 2006; Claassen et al. 2007; Kwan et al. 2011; Allan et al. 2013). Several of these studies show a low proportion of trauma recidivism, which is probably due to the facts that they only include serious injuries or only patients handled by the specific trauma team at the hospital. Moreover, their follow- up was shorter. And also their patients may visit another hospital if injured again. In our study there was just one trauma hospital in the area. Consequently, we claim that many of these studies does not reflect the full burden of injury recidivism. The high proportion of trauma recidivism in our material indicates a significant opportunity for preventive intervention.

### Age and sex

We found that young men often suffered proportionately more recurrent injuries than young women. This has also been shown by Kaufmann (Kaufmann et al. 1998). In the elderly population, we found that women were more affected by recurrent injuries, which is consistent with older previously published results, but in contrast to a recently published study from a trauma center in Miami, which found more men among recurrently injured over 65 (Gubler et al. 1996; Allan et al. 2013). In this study, they had 20 years follow up, but found only 1.2 % recurrent injuries, perhaps due to a higher migration rate. This study was done in a major urban trauma center and their recidivists were more susceptible to penetrating trauma, all-terrain vehicle/motorcycle collisions and possibly bicycle accidents, which indicates a completely different case mix compared to our study. Most previous studies have not analyzed the recidivism from an age perspective. Our results show that both age and sex are important factors that affect the risk of recurrent injury.

### Type of injury

Fracture and sprain increased the risk for moderate recurrent injury. Clinical examples of sprains are anterior cruciate ligaments injuries, which affect the stability and muscular strength of the knee and anterior dislocation of the shoulder that at younger ages often leads to recurrent dislocation (Cutts et al. 2009; Gardinier et al. 2012). Consequently, the increased risk may be caused by impairment of the functional level of extremities. Wounds and foreign body penetration on the other hand were type of injuries that lowers the risk for future moderate or serious injury. These injuries are likely to heal without compromising the physical function. A Baltimore study with mostly younger trauma patients and a Miami study with elderly both reported that recidivists were more often injured by penetrating trauma the first time (Brooke et al. 2006; Allan et al. 2013). This is in contrast to a study from Nevada, where blunt trauma was more common (Toschlog et al. 2007). Again, we believe that the difference between our results and the results in these studies can be explained by differences in case mix, where our study includes more mild and moderate injuries. That concussion as a first injury increase the risk for subsequent injury is known in sport medicine, in elite football players and in in ice-hockey, football, floorball and hand-ball but has to our knowledge not been shown for a wider population (Nordstrom et al. 2014; Nyberg et al. 2015). To our knowledge this is the first study to show this association in a wider population. Earlier studies have indicated that concussion may compromise cognitive function in the short time interval, which could enhance the risk for subsequent injury (Shores et al. 2008; Peterson et al. 2009). Burman’s study on the other hand indicated that athletes who suffer a concussion might be more injury prone due to a higher willingness to take risks (Burman et al. 2016).

So far, no other study like this one, populations based and longitudinal over 14 years, has previously investigated the type of first time injury as a possible indicator for recurrent injury. Our results show that fractures, sprains and concussion can have an impact on future injury and thus may be a variable to use when selecting individuals for prevention programs.

### Level of injury severity

More severe injuries increased the risk for recurrent moderate injury, which is in line with studies on trauma recidivism among the elderly, but in contrast to other studies (Hedges et al. 1995; Gubler et al. 1996). The higher risk after more severe injuries might be explained by a persistent more severe impairment after injury, which affects the risk for subsequent injury (McGwin et al. 2001). However, there might also be behavioral factors contributing to make some individuals more injury prone than others.

### Strengths and limitations

The strength of this study is that it provides information for a complete population in a well-defined, stable geographic area with just one hospital. Moreover, during this period no major reorganizations within the health care system were implemented. The database on which this study was based has a high degree of validity and coverage.

A drawback is the selection bias. We define the first injury in the registry (1999-) as the subject’s first injury although they may have suffered one or more injuries before 1999. In addition, a few fatal injuries (immediate deaths at the site of accident) were not included in the study. Both those conditions will have a diluting effect on our results. Some variables. Which have been associated with trauma recidivism like socioeconomic status, chronic disease and use of drugs, were not included in this study.

## Conclusions

Trauma recidivism is common and contributes to a considerable part of the trauma burden, which emphasize a need for prevention. Injury type, sex and age can be used as variables in the selection of participants for preventive programs. An acute accident is a pedagogic opportunity. Individual information that the risk for future injury is elevated may motivate the patient to participate in a preventive program.
